# Supplementary material for: Benchmarking the geographic generalization of deep learning models for precipitation downscaling
Source: Sci Rep. 2026 Jan 27;16:3733. doi: 10.1038/s41598-025-34557-4 (PMC12852169; doi:10.1038/s41598-025-34557-4)
Supplement: Supplementary file 1 — Supplementary Information. [file 41598_2025_34557_MOESM1_ESM.pdf]

Supplementary information

Model	Training Areas	E1 Cape Horn	E2 Amazon Basin	E3 West Africa	E4 Horn of Africa	E5 Tibetan Plateau	E6 Melanesia
		Target Areas					
Mean precip.	ERA5	0.120	0.183	0.071	0.075	0.045	0.318
	IMERG	0.100	0.184	0.083	0.069	0.025	0.293
Max precip.	ERA5	29.269	46.221	57.219	63.488	58.945	63.135
	IMERG	73.730	125.715	122.400	132.415	85.315	152.550
Std precip.	ERA5	0.376	0.630	0.437	0.420	0.198	0.784
	IMERG	0.489	0.866	0.685	0.539	0.236	1.239
P99 precip.	ERA5	1.857	3.047	1.752	1.640	0.880	3.403
	IMERG	2.235	4.155	2.410	1.980	0.640	5.480
P0 precip.	ERA5	0.730	0.713	0.878	0.830	0.858	0.490
	IMERG	0.866	0.863	0.941	0.938	0.946	0.767
Wet intensity	ERA5	0.432	0.629	0.569	0.429	0.305	0.617
	IMERG	0.744	1.345	1.403	1.115	0.468	1.256

Table S1. Descriptive statistics. Values in [mm/h] for ERA5 and IMERG precipitation across target areas.

Variable	Description
tp	Total precipitation
cp	Convective precipitation
cape	Convective potential energy
twc	Total water content
tlwc	Total liquid water content
sp	Surface pressure
tisr	Top-of-the-atmosphere incident solar radiation
u	Eastward wind velocity at 700 hPa
v	Northward wind velocity at 700 hPa

Table S2. List of atmospheric variables used as predictors.

Model	Training Areas	Target Areas<br>E1<br>Cape Horn	 E2<br>Amazon Basin	 E3<br>West Africa	 E4<br>Horn of Africa	 E5<br>Tibetan Plateau	 E6<br>Melanesia
ERA5 Interp.	-	1.955	3.932	2.005	1.361	0.632	5.658
GAN	A_1	**1.210**	3.003	1.708	**1.133**	**0.474**	4.827
DM	A_1	1.839	**1.610**	**1.059**	1.276	0.528	5.334
ResNet	A_1	1.490	3.801	1.919	1.345	0.602	**4.713**
GAN	A_2	0.999	2.225	1.673	0.773	0.563	2.436
DM	A_2	**0.899**	**0.765**	**0.460**	**0.532**	**0.425**	**2.292**
ResNet	A_2	1.443	3.658	1.923	1.339	0.598	3.998
GAN	A_3	0.992	3.017	1.679	1.081	**0.280**	4.608
DM	A_3	**0.800**	**0.982**	**0.894**	**0.582**	0.409	**1.006**
ResNet	A_3	1.258	3.678	1.933	1.351	0.618	4.488
GAN	A_4	**0.634**	1.536	1.130	0.661	**0.251**	2.878
DM	A_4	0.771	**1.144**	**0.697**	**0.494**	0.450	**1.827**
ResNet	A_4	1.258	3.678	1.933	1.351	0.618	4.488
GAN	E_i	1.352	1.081	1.706	1.703	0.301	3.558
DM	E_i	0.716	0.534	0.534	1.104	0.432	1.559
ResNet	E_i	0.966	3.782	1.923	1.334	0.617	3.784

Table S3. Accuracy of models on spatial generalization tasks. Test 99th percentile MAE (lower better) for precipitation in mm/h on the designated evaluation area and averaged over test years 2021 – 2022. Best scores per subtask are in bold. The last three rows (E_i) are not contestants in the benchmark but show what is possible when training directly on the target.

Model	Training Areas	Target Areas<br>E1<br>Cape Horn	 E2<br>Amazon Basin	 E3<br>West Africa	 E4<br>Horn of Africa	 E5<br>Tibetan Plateau	 E6<br>Melanesia
ERA5 Interp.	-	0.447	1.005	0.252	0.163	0.065	1.716
GAN	A_1	**0.259**	0.864	0.212	**0.142**	**0.052**	**1.457**
DM	A_1	0.439	**0.587**	**0.115**	0.163	0.062	1.676
ResNet	A_1	0.358	0.984	0.243	0.163	0.062	1.567
GAN	A_2	0.303	0.781	0.190	0.109	0.075	**0.756**
DM	A_2	**0.274**	**0.331**	**0.139**	**0.107**	0.095	0.798
ResNet	A_2	0.369	0.987	0.248	0.162	**0.063**	1.436
GAN	A_3	**0.222**	0.779	0.209	**0.125**	**0.054**	1.330
DM	A_3	0.286	**0.537**	**0.153**	0.132	0.056	**0.481**
ResNet	A_3	0.320	0.989	0.249	0.163	0.065	1.541
GAN	A_4	**0.144**	**0.339**	0.090	**0.080**	**0.052**	**0.753**
DM	A_4	0.249	0.543	**0.107**	0.093	0.075	0.895
ResNet	A_4	0.381	0.992	0.248	0.163	0.065	1.493
GAN	E_i	0.295	0.417	0.200	0.080	0.039	0.981
DM	E_i	0.128	0.334	0.146	0.156	0.058	0.672
ResNet	E_i	0.221	0.992	0.248	0.162	0.064	1.471

Table S4. Accuracy of models on spatial generalization tasks. Test 95th percentile MAE (lower better) for precipitation in [mm/h] on the designated evaluation area and averaged over test years 2021 – 2022. Best scores per subtask are in bold. The last three rows (E_i) are not contestants in the benchmark but show what is possible when training directly on the target.

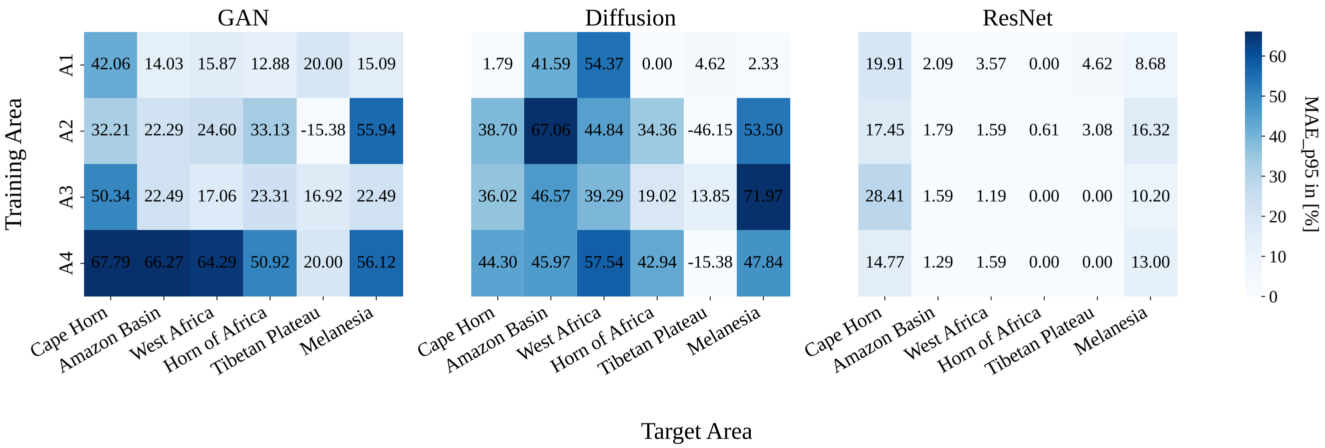

Figure S1. Heatmap of % improvement relative to interpolation. Change in 95th percentile MAE (lower better) in [%] for each model relative to bilinear interpolation.

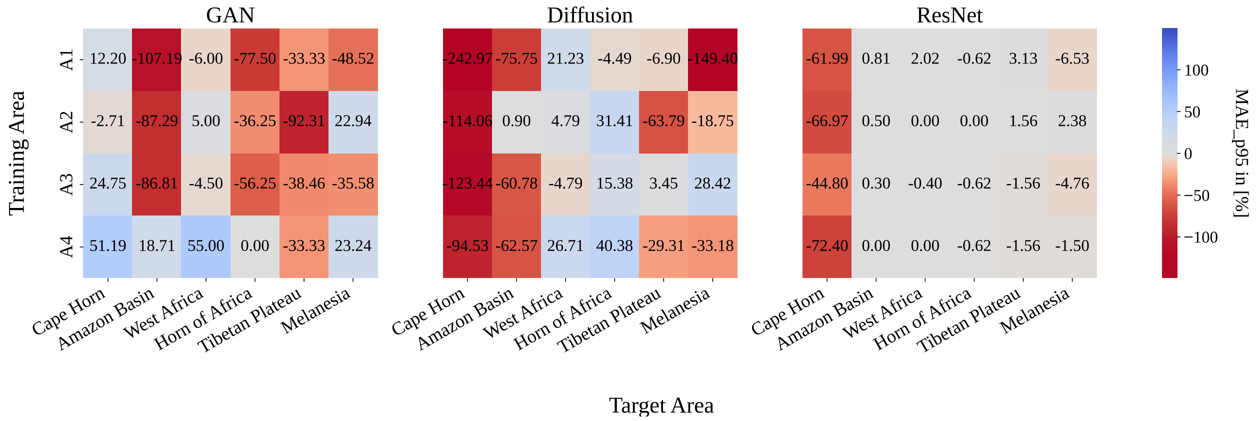

Figure S2. Heatmap of % performance drop between in and out-of-distribution training. Change in 95th percentile MAE (lower better) in [%] for each model relative to training the model directly on the target regions.
